# Corylin Ameliorates LPS-Induced Acute Lung Injury via Suppressing the MAPKs and IL-6/STAT3 Signaling Pathways

**DOI:** 10.3390/ph14101046

**Published:** 2021-10-14

**Authors:** I-Chen Chen, Shu-Chi Wang, Yi-Ting Chen, Hsin-Han Tseng, Po-Len Liu, Tzu-Chieh Lin, Hsin-En Wu, Yuan-Ru Chen, Yu-Hsin Tseng, Jong-Hau Hsu, Zen-Kong Dai, Jau-Ling Suen, Chia-Yang Li

**Affiliations:** 1 Department of Pediatrics, Kaohsiung Medical University Hospital, Kaohsiung 80756, Taiwan; yljane.chen@gmail.com (I.-C.C.); grapepuff@gmail.com (Y.-H.T.); jhh936@yahoo.com.tw (J.-H.H.); zenkong@gmail.com (Z.-K.D.); 2 Department of Pediatrics, School of Medicine, College of Medicine, Kaohsiung Medical University, Kaohsiung 80708, Taiwan; 3 Graduate Institute of Medicine, College of Medicine, Kaohsiung Medical University, Kaohsiung 80708, Taiwan; yt0728@gmail.com (Y.-T.C.); warrior-215@hotmail.com (H.-H.T.); 990327kmuh@gmail.com (T.-C.L.); angxxie@gmail.com (H.-E.W.); yuannns90@gmail.com (Y.-R.C.); 4 Department of Medical Laboratory Science and Biotechnology, Kaohsiung Medical University, Kaohsiung 80708, Taiwan; shuchiwang@kmu.edu.tw; 5 Department of Pathology, Kaohsiung Medical University Hospital, Kaohsiung Medical University, Kaohsiung 80708, Taiwan; 6 Department of Pathology, Faculty of Medicine, College of Medicine, Kaohsiung Medical University, Kaohsiung 80708, Taiwan; 7 Department of Respiratory Therapy, College of Medicine, Kaohsiung Medical University, Kaohsiung 80708, Taiwan; kisa@kmu.edu.tw; 8 Division of Cardiology, Department of Internal Medicine, Kaohsiung Medical University, Kaohsiung 80708, Taiwan; 9 Research Center for Environmental Medicine, Kaohsiung Medical University, Kaohsiung 80708, Taiwan; 10 Department of Medical Research, Kaohsiung Medical University Hospital, Kaohsiung 80756, Taiwan; 11 Center for Cancer Research, Kaohsiung Medical University, Kaohsiung 80708, Taiwan

**Keywords:** acute lung injury, acute respiratory distress syndrome, corylin, IL-6, TNF-α, STAT3, MAPK signaling pathway

## Abstract

Acute lung injury (ALI) is a high mortality disease with acute inflammation. Corylin is a compound isolated from the whole plant of *Psoralea corylifolia* L. and has been reported to have anti-inflammatory activities. Herein, we investigated the therapeutic potential of corylin on lipopolysaccharides (LPS)-induced ALI, both in vitro and in vivo. The levels of proinflammatory cytokine secretions were analyzed by ELISA; the expressions of inflammation-associated proteins were detected using Western blot; and the number of immune cell infiltrations in the bronchial alveolar lavage fluid (BALF) were detected by multicolor flow cytometry and lung tissues by hematoxylin and eosin (HE) staining, respectively. Experimental results indicated that corylin attenuated LPS-induced IL-6 production in human bronchial epithelial cells (HBEC3-KT cells). In intratracheal LPS-induced ALI mice, corylin attenuated tissue damage, suppressed inflammatory cell infiltration, and decreased IL-6 and TNF-α secretions in the BALF and serum. Moreover, it further inhibited the phosphorylation of mitogen-activated protein kinases (MAPKs), including p-JNK, p-ERK, p-p38, and repressed the activation of signal transducer and activator of transcription 3 (STAT3) in lungs. Collectively, our results are the first to demonstrate the anti-inflammatory effects of corylin on LPS-induced ALI and suggest corylin has significant potential as a novel therapeutic agent for ALI.

## 1. Introduction

Acute lung injury (ALI) and/or acute respiratory distress syndrome (ARDS), one of the major causes of mortality and morbidity in intensive care, has a great impact on public health [1,2,3]. For years, the most significant advance in the management of ALI/ARDS concerns protective mechanical ventilation strategies, although no pharmacological intervention has been shown to be effective [4,5]; thus, it is urgent to develop novel potential pharmaceutical drugs for the management of ALI/ARDS.

The hallmark of ALI/ARDS is that injury to the epithelium results in the release of inflammatory mediators, promoting the initial influx of neutrophils and macrophages into the sites of injury, following an increase in cytokine production and flooding of protein-rich fluid into the alveolar space [3,6]. The infiltration of inflammatory cells leads to an excessive inflammatory response, involving a complex group of mediators such as proinflammatory cytokines, interleukin (IL)-1β, tumor necrosis factor (TNF)-α, IL-6, and IL-8 [5,7]. In the lungs, it has been shown that IL-6 plays an essential role in phosphorylation of signal transducer and activator of transcription 3 (STAT3), resulting in enhancing neutrophil recruitment and decreasing bacterial burdens [8,9,10]. In addition, other signal transduction pathways that participate in mediating lung inflammation include JAK/STAT, NF-κB, and mitogen-activated protein kinase (MAPK) signal transduction [10,11,12,13].

Corylin, a compound isolated from the fruit of *Psoralea corylifolia* L., has been reported to have multiple biological activities such as anti-osteoclastic, anti-obesity, anti-oxidative, anti-inflammatory, and anti-tumor effects [14,15,16,17,18,19,20,21]. In terms of the anti-inflammatory effects, corylin can inhibit TNFα-induced monocyte adhesion by suppressing ROS production, MAPK phosphorylation and NF-κB p65 translocation [15]. Corylin can also inhibit LPS-induced inflammatory responses, including nitric oxide (NO), inducible NO synthase (iNOS), and other proinflammatory cytokines (TNF-α, IL-6, and IL-1β) through the activation of both MAPKs and NLRP3 inflammasome pathways [16]. Moreover, corylin also has protective effects in LPS-induced septic shock [17]. Notably, corylin exhibits potent anti-inflammatory activity on IL-6-stimulated hepatocarcinoma cells through suppressing IL-6-induced phosphorylation of STAT3 [22]. Based on this evidence, we hypothesized that corylin might have protective effects on LPS-induced ALI; therefore, in this study, we aimed to examine the anti-inflammatory effects of corylin on LPS-induced ALI both in vitro and in vivo.

## 2. Results

### 2.1. Corylin Suppresses the Production of IL-6 by LPS-Induced Human Bronchial Epithelial Cells (HBEC3-KT Cells)

**Figure 1:**
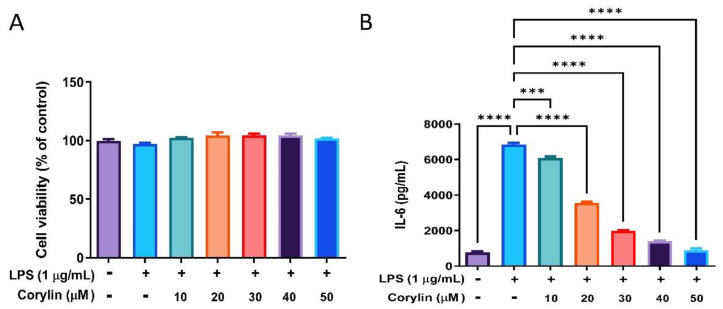
The effects of corylin on the cell viability and the production of IL-6 by LPS-induced HBEC3-KT cells. Cells were pre-treated with different concentrations of corylin (0~50 μM) for 1 h following treatment of LPS (1 μg/mL) for 24 h. (**A**) Cell viability was examined by MTT assay. (**B**) The concentration of IL-6 in the cell culture supernatant was detected using ELISA. The data are presented as means ± SEM of three independent experiments. Statistical significances are represented as follows: *** *p* < 0.001 and **** *p* < 0.0001 vs. LPS alone.

At first, the effect of corylin on the cell viability of HBEC3-KT cells were examined using MTT assay. Cells were pre-treated with various concentrations of corylin (10–50 μM) for 1 h following treatment of LPS (1 μg/mL) for 24 h. As shown in Figure 1A, no cytotoxic effect was revealed when the HBEC3-KT cells were treated with corylin ≤ 50 μM. IL-6 is one of the major proinflammatory cytokines in ALI. The effects of corylin on IL-6 production by LPS-induced HBEC3-KT cells was therefore examined, and experimental results showed that corylin significantly suppressed the production of IL-6 by LPS-induced HBEC3-KT cells in a concentration-dependent manner (Figure 1B). Other pro-inflammatory cytokines were also checked, including TNF-α, IL-1β and IL-12, as well as nitric oxide production; however, none of these were detected in supernatant of LPS-induced HBEC3-KT cells (data not shown).

### 2.2. Corylin Attenuates the Production of Inflammatory Cytokines in LPS-Induced ALI Mice

**Figure 2:**
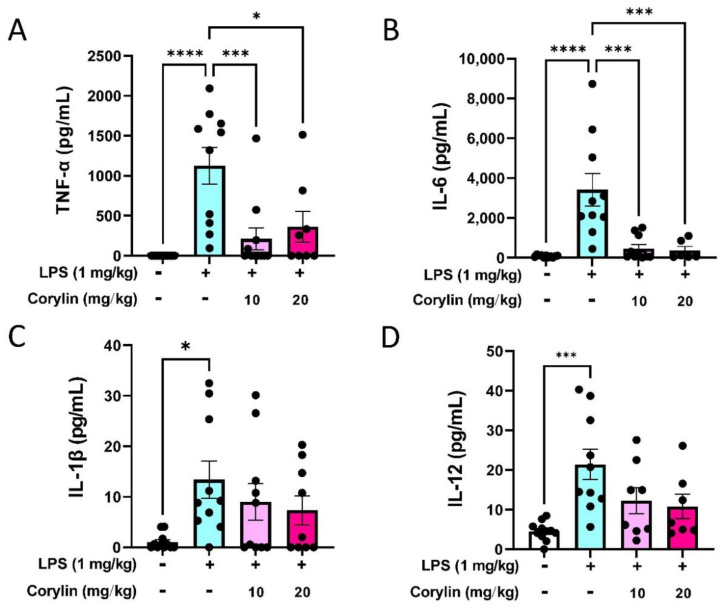
Effects of corylin on LPS-induced inflammatory cytokine productions in BALF. Mice were intraperitoneally injected with corylin (10 mg/kg or 20 mg/kg) for 1 h following intratracheal administration of LPS for 4 h. The mice were sacrificed and the BALF was collected. The expression levels of (**A**) TNF-α, (**B**) IL-6, (**C**) IL-1β, and (**D**) IL-12 in BALF were analyzed using ELISA. The data are presented as means ± SEM of three independent experiments. Statistical significances are represented as follows: * *p* < 0.05, and *** *p* < 0.001 and **** *p* < 0.0001 vs. LPS alone.

To access the effects of corylin on the production of inflammatory cytokines in LPS-induced ALI mice, concentrations of TNF-α, IL-6, IL-1β, and IL-12 in bronchial alveolar lavage fluid (BALF) were measured by ELISA. After LPS administration, the levels of TNF-α, IL-6, IL-1β, and IL-12 in BALF were significantly increased as compared to the PBS-treated control group (Figure 2). In particular, treatment with corylin significantly reduced TNF-α and IL-6 production in BALF, compared with those in the LPS group (Figure 2A,B). However, the levels of IL-1β and IL-12 had decreasing trend but with no statistical significance in the presence of corylin (Figure 2C,D). Overall, these data suggested that corylin could partially suppress the production of inflammatory cytokines in BALF in LPS-induced mice.

### 2.3. Corylin Reduces the Infiltration of Inflammatory Cells in Lung in LPS-Induced ALI Mice

**Figure 3:**
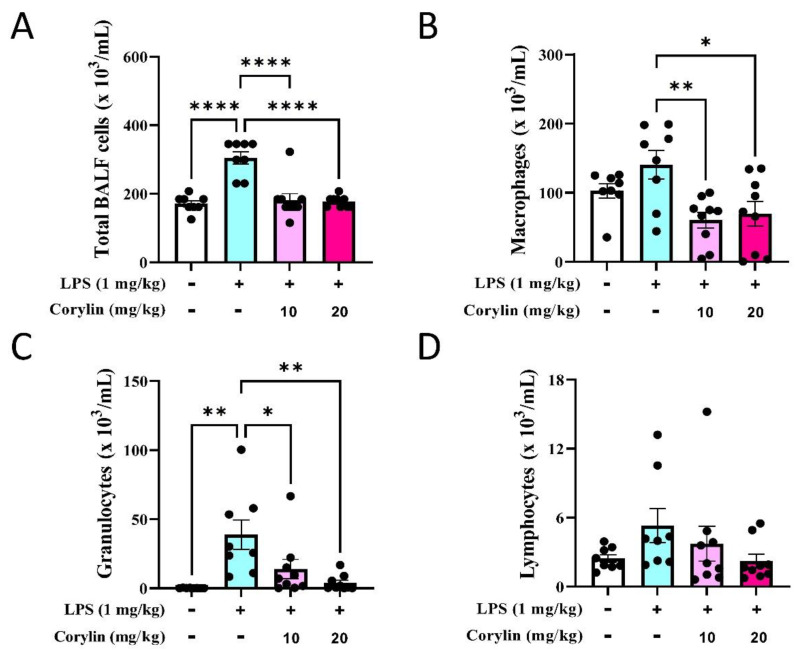
Effects of corylin treatment on immune cell infiltration in BALFs in LPS-induced mice. BALFs were collected from LPS and/or corylin-treated mice. (**A**) The total cell numbers in BALFs. The cell subsets were identified by multi-color flow cytometry, including (**B**) macrophages, (**C**) granulocytes, and (**D**) lymphocytes. Results are shown as mean ± SEM. * *p* < 0.05, ** *p* < 0.01, and **** *p* < 0.0001 vs. LPS alone by one-way ANOVA followed by post hoc Tukey’s test. The numbers of mice are pooled from two independent experiments (*n* = 8~10).

Since the number of inflammatory cell infiltrations is a hallmark of ALI, we next explored whether corylin treatment could suppress LPS-induced lung inflammation. The flow cytometric analysis showed that intratracheal LPS instillation recruited significantly immune cell infiltration in BALFs, including macrophages and granulocytes (Supplemental Figure S1 and Figure 3A–C). The low cell numbers of lymphocytes recruited in BALFs (Figure 3D) may be due to the short-time treatment of LPS. In support of anti-inflammatory activity, corylin/LPS-treated mice exhibited significantly less lung inflammation compared to the LPS-treated group; in particular, with decreased numbers of macrophages and granulocytes in BALFs (Figure 3). This result indicated that corylin has anti-inflammatory activity in LPS-induced inflammation.

### 2.4. Corylin Decreases the Phosphorylation of MAPKs and STAT3 in Lung Tissues of LPS-Induced ALI Mice

**Figure 4:**
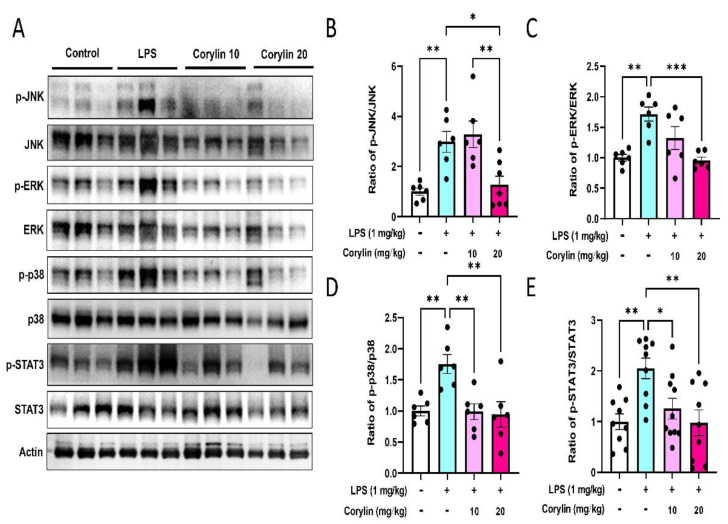
Effects of corylin on the expression and phosphorylation of MAPKs and STAT3 in lung tissues. Expressions of phospho-JNK, JNK, phospho-ERK, ERK, phospho-p38 MAPK, p38 MAPK, phospho-STAT3 and STAT3 were analyzed by Western blot. (**A**) The representative blot of triplicate experiments. Quantitated results of (**B**) phospho-JNK/JNK ratio (*n* = 6), (**C**) phospho-ERK/ERK ratio (*n* = 6), (**D**) phospho-p38/p38 ratio (*n* = 6), and (**E**) phospho-STAT3/STAT3 ratio (*n* = 9~10) were shown as mean ± SEM. The relative fold of phosphorylation activity was normalized to untreated samples. (* *p* < 0.05, ** *p* < 0.01 and *** *p* < 0.001 vs. LPS alone).

MAPKs and STAT3 are the key signaling regulators in modulating the production of pro-inflammatory mediators and cytokines during infection [23,24]. In order to explore the molecular mechanism responsible for inhibiting proinflammatory cytokine production and inflammatory cell infiltration by corylin treatment, Western blot analysis was performed to examine the expression of MAPKs and STAT3 in lung tissues. As revealed in Figure 4, intratracheal administration of LPS significantly increased the phosphorylation of JNK, ERK, and STAT3 in lung tissue (Figure 4A–E). While the expression of phosphorylation of p38 MAPK had an increasing trend in LPS-induced mice compared with the control group, this did not reach statistical significance (Figure 4D). In addition, experimental results indicated that corylin (10 and 20 mg/kg) treatment significantly decreased the phosphorylation of JNK, ERK, p38, and STAT3 (Figure 4).

### 2.5. Corylin Reduces the Expression of IL-6 in Mouse Serum

**Figure 5:**
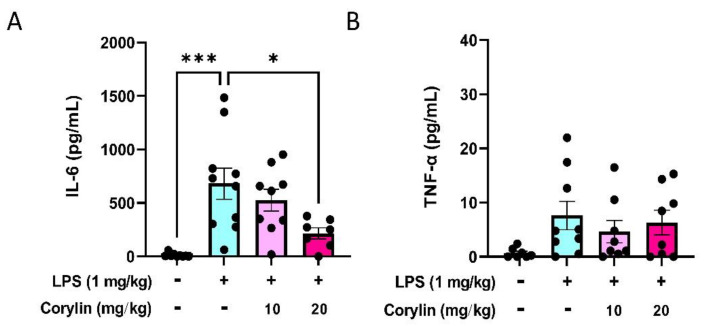
Effects of corylin on the expression of IL-6 and TNF-α in serum of LPS-induced mice. The expression levels of (**A**) IL-6 and (**B**) TNF-α in serum were measured using ELISA. The data are shown as mean ± SEM (*n* = 7~9). (* *p* < 0.05 and *** *p* < 0.001 vs. LPS alone).

To examine the effect of corylin on the secretion of proinflammatory cytokines in circulation, the serum levels of IL-6 and TNF-α were examined by ELISA. As shown in Figure 5, the level of IL-6 was significantly increased in serum compared with the control group after intratracheal LPS stimulation for 4 h, whereas corylin treatment significantly reduced the secretion of IL-6 in a dose-dependent manner (Figure 5A). However, the level of TNF-α in serum was not increased after intratracheal LPS stimulation and the level of TNF-α revealed no significant difference in either LPS or LPS plus corylin treatments (Figure 5B).

### 2.6. Corylin Decreases Lung Injury in LPS-Induced ALI Mice

**Figure 6:**
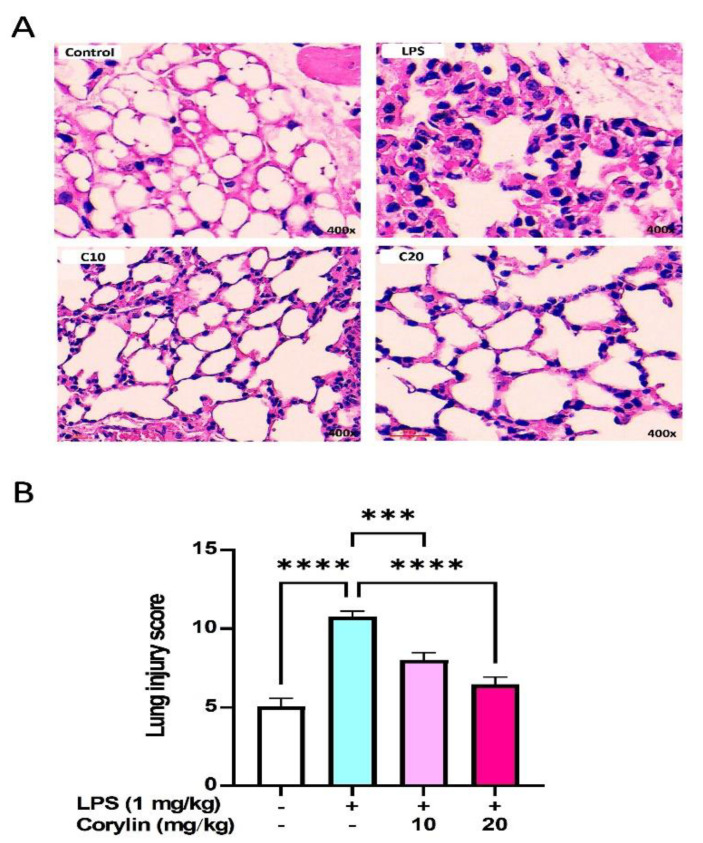
Effects of corylin on the lung in LPS-challenged mice. (**A**) Representative photographs of the lung tissues stained with HE. Upper left, control, the representative image PBS control. Upper right, the representative image of LPS administration. Lower left, the representative image of low dose corylin (10 mg/kg) pre-treatment following LPS administration. Lower right, the representative image of high dose corylin (20 mg/kg) pre-treatment following LPS administration. (**B**) Morphological changes in lung sections were semi-quantified using lung injury score. The results showed a significant reduction in the severity of lung injury in mice treatment with corylin compared to the LPS-induced ALI mice (*n* = 4~6). The magnification is 400X. The data are shown as mean ± SEM. (*** *p* < 0.001 and **** *p* < 0.0001 vs. LPS alone).

To further examine the effect of corylin on the histological changes in LPS-induced ALI mice, the HE staining method was employed. As shown in Figure 6A, a large number of inflammatory cells around the alveoli were observed in LPS-stimulated mice, whereas corylin treatment obviously attenuated inflammatory cell infiltration in the lungs; moreover, the ALI score was higher in the LPS group compared to the control group, whereas corylin treatment significantly decreased the ALI score (Figure 6B).

## 3. Discussion

The present study firstly demonstrates that corylin, a main compound isolated from *Psoralea corylifolia* L., has protective effects against LPS-induced ALI, both in vitro and in vivo. To illustrate the effect of corylin on ALI, a series of assays in ALI mice with intratracheal administration of LPS were performed, and as expected, corylin reduced inflammatory cell infiltration in lung tissue and attenuated lung injury in LPS-induced ALI mice. LPS administration induced an acute inflammatory response through increasing proinflammatory cytokine secretions in BALF and serum, while corylin treatment significantly inhibited the production of proinflammatory cytokine in both BALF and serum as well as significantly inhibiting the phosphorylation of JNK, ERK, p38, and STAT3 in lung tissue. Additionally, in HBEC3-KT cells, pre-treatment with corylin markedly inhibited the production of IL-6 by LPS-induced HBEC3-KT cells. Collectively, these experimental results illustrate that corylin had protective effects against LPS-induced ALI through inhibition of MAPKs and IL6/STAT3 signal pathways.

Macrophages play important roles in host defense to infection, repair of damaged tissue, and secretion of pro-inflammatory cytokines such as TNF-α and IL-6 to modulate inflammatory response [25]. In the lung, alveolar macrophages are responsible for the recruitment of neutrophils from the vascular space to the airspace when LPS is administrated by intra–alveolar routes [26]. An interesting finding showed the cell density of macrophages in BALF was almost the same level in both LPS-induced and PBS-administrated (control) mice (Figure 3B). We speculated that physical injury by intratracheal administration of PBS might also recruit residential macrophage release from the lung into the alveolar space; however, alveolar macrophage recruitment might not necessarily induce the secretion of proinflammatory cytokines since these cytokines were not elevated in PBS-administrated mice in our study. In line with other previous studies, alveolar macrophages regulated neutrophil recruitment and did not play a critical functional role as neutrophils do in acute endotoxin-induced lung injury [27,28].

The influx of inflammatory cells and the release of inflammatory mediators in the lung is a main characteristic of ALI [5]. The accumulation of neutrophils in lung microvasculature, interstitial and bronchoalveolar space is believed to play a key role in ALI/ARDS [29]. Neutrophils secrete potent antibacterial molecules including protease, cationic compounds, and reactive oxidants; however, migration neutrophils also lead to mechanical damage to the alveolar lumen and further worsen the influx of fluid into the alveolar space [29,30]. In this study, it was found that LPS induced significant acute inflammatory signs in lung tissues and a large increase of inflammatory cells in BALF, consistent with the experimental observations that inflammatory cell infiltration is the hallmark in ALI induced by LPS [31,32]. According to our experimental results, corylin attenuated LPS-induced acute inflammatory signs in lung tissue and suppressed the recruitment of inflammatory cells in BALF of LPS-induced ALI mice. Taken together, these results demonstrated that corylin has protective benefits in LPS-induced ALI.

Except for inflammatory cell infiltration, the release of proinflammatory cytokines, especially IL-1β, TNF-α, and IL-6, has been reported to be profoundly involved in the inflammatory cascade of LPS-induced ALI [5]. These cytokines are also predictive of the outcome of ALI clinically [5,33]. Apart from these markers, IL-12, classified as part of the IL-6/IL-12 family, has been considered as a key immunoregulatory cytokine that contributes to T-cell differentiation and coordinates innate and adaptive immune systems [34,35]. In the present study, the releases of IL-1β, TNF-α, IL-6, and IL-12 were markedly induced by LPS challenge, whereas treatment with corylin obviously reduced LPS-induced IL-6 and TNF-α production in BALF and IL-6 in serum. Expressions of both IL-1β and IL-12 in BALF did not reach statistical significance after corylin treatment; however, they still showed a deceasing trend under corylin treatment. Moreover, in line with other previous studies, the bronchial epithelial cells could secrete IL-6 and other proinflammatory cytokines under the LPS-stimulation [36,37], and in our experiment, the administration of corylin majorly blocked the production of IL-6 in HBEC3-KT cells.

The MAPK pathways include JNK, p38 MAPK and ERK pathways, which play important roles in triggering pro-inflammatory cytokine and mediator production in response to stimulation of LPS [38,39,40]. Of note, not limited to MAPKs, STAT3 tyrosine phosphorylation is also important in IL-1β and IL-6 production in response to inflammation [41]. Our results showed that intratracheal administration of LPS obviously induced the expression of MAPKs and SAT3 in lung tissue, and corylin significantly inhibited both LPS-induced MAPKs and STAT3 activation. Consistent with the production of inflammatory cytokines (TNF-α and IL-6), we suggest that corylin exhibits protective effects on ALI by inhibiting MAPKs and STAT3 activation.

There are two limitations presented in this study. Firstly, HBEC3-KT cells lacked several inflammatory characteristics, including non-secretion of TNF-α, IL-1β and IL-12 under LPS treatment; therefore, this cell line might not be suitable for further mechanistic study. Secondly, gender difference was not investigated in the present study since sex differences might have different susceptibilities to pathogens, resulting in distinct levels of immune responses [42]. In mice, Klein and Flanagan have reported that the pro-inflammatory cytokine responses, T cell proliferation and antibody responses are greater in females than in males [42]; however, in murine ALI, male mice had more lung oedema, protein leaks and histological evidence of injury than female mice [43]. Whether gender difference affects the anti-inflammatory properties of corylin on ALI needs to be further clarified.

## 4. Materials and Methods

### 4.1. Mice

Female C57BL/6 mice (ages 6–8 weeks, weighing 17–19 g) were purchased from the National Lab Animal Center (Taipei, Taiwan), with the experimental protocol for all mice being approved by the Committee on the Ethics of Animal Experiments of the Kaohsiung Medical University (Permit Number: 108081, approval date: 1 November 2019~31 October 2022). A total of sixty mice were used and divided into four groups, each containing fifteen mice. The animals were housed in a temperature-controlled room with a 12-h day/night cycle at 25 ± 1 °C and supplied with food and water in a constant environment. All animal works were performed in an Association for Assessment and Accreditation of Laboratory Animal Care International (AAALAC)-accredited facility.

### 4.2. Cell Culture

A human bronchial epithelial cell line, HBEC3-KT cells, was purchased from American Type Culture Collection (No. CRL-4051, Manassas, VA, USA). Cells were cultured in Ham’s F-12K medium supplemented with 10% fetal bovine serum and 1% penicillin and streptomycin (Corning, Corning, NY, USA), and incubated in a humidified atmosphere of 5% CO_2_ at 37 °C.

### 4.3. Cell Viability Assay

The MTT assay was carried out in the HBEC3-KT cells to measure the cytotoxic effect of corylin (ChemFaces, Wuhan, Hubei, China). HBEC3-KT cells were seeded at a concentration of 5 × 10^4^ cells/mL into a 96-well plate. Following overnight adherence, the cells were pre-treated with 0–50 μM corylin (purchased from ChemFaces, Wuhan, Hubei, China; purity > 98%; dissolved in dimethyl sulfoxide (DMSO)) to produce a stock solution and serial dilutions were prepared by phosphate-buffered saline (PBS), and then treated with LPS (1 μg/mL) for 24 h. Then, 10 μL MTT solution (5 mg/mL in PBS, Sigma Aldrich, St. Louis, MO, USA) was added to each well and mixed. After 4 h, the medium was then aspirated, and the formed formazan crystals were solubilized by adding 100 μL acidified isopropyl alcohol (0.04 N HCl). The intensity of the dissolved formazan crystals (purple color) was quantified using the microplate reader at 570 nm.

### 4.4. ALI Model

Mice were randomly divided into four groups: a control group, 1 mg/kg LPS group, a 10 mg/kg corylin + 1 mg/kg LPS group, and a 20 mg/kg corylin + 1 mg/kg LPS group. The mice were intraperitoneally (i.p.) injected with corylin for 1 h following intratracheal (i.t.) LPS instillation for 4 h, with the control group being administrated with PBS instead of corylin and LPS. All mice were humanely sacrificed 4 h after LPS treatment; afterwards, the bronchoalveolar lavage fluid (BALF) and lung tissues were collected for subsequent analysis.

### 4.5. Enzyme-Linked Immunosorbent Assay (ELISA)

Cell culture supernatants, murine bronchioalveolar lavage fluid (BALF) and murine serum were analyzed for TNF-α, IL-1β, IL-6, and IL-12 using ELISA kits (Thermo Scientific, Waltham, MA, USA) following the manufacturer’s instructions.

### 4.6. Multicolor Flow Cytometry

The staining panel for immune cell subsets in BALF was designed according to previous studies [44,45]. Briefly, the BALF cells were stained with fluorochrome-conjugated monoclonal antibodies at 4 °C for 30 min. The antibodies included FITC conjugated anti-Ly6G (1A8; BD Biosciences, San Jose, CA, USA), PE-conjugated anti-Siglec-F (E50-2440; BD Biosciences), APC-conjugated anti-B220 (RA3-6B2; BD Biosciences), APC-conjugated anti-CD3 (145-2C11; BD Biosciences), PerCP/Cy5.5-conjugated anti-CD11b (M1/70; BioLegend, San Diego, CA, USA), eFluor 450-conjugated anti-CD11c (N41B; Invitrogen, Eugene, OR, USA), and Live/Dead fixable Red (Invitrogen). After washing, the samples were analyzed with multi-parametric flow cytometry (LSR II; BD Boisciences) and data were analyzed with FlowJo software (version 10, Tree Star, Inc., Ashland, OR, USA).

### 4.7. Western Blot Analysis

Lung tissues were homogenized in RIPA buffer containing protease inhibitors and phosphatase inhibitors (Sigma Aldrich) and centrifuged at 12,000× *g* for 10 min. Supernatants were collected and total protein levels were measured using BCA protein assay (Thermo Scientific). Proteins were separated by SDS-PAGE and transferred to polyvinylidene fluoride membranes (Millipore Corporation, Billerica, MA, USA), with protein expression levels being analyzed using antibodies against phospho-extracellular-signal-regulated kinase (ERK)1/2 (CST#4370, Cell Signaling, Farmingdale, NY, USA), ERK1/2 (CST#4695, Cell Signaling), phospho-JUN N-terminal kinase (JNK) 1/2 (CST#9255, Cell Signaling), JNK 1/2 (CST#9258, Cell Signaling), phospho-p38 MAPK (CST#4511, Cell Signaling), p38 MAPK (CST#8690, Cell Signaling), phospho-STAT3 (CST#9145, Cell Signaling), STAT3 (CST#9139, Cell Signaling), and β-actin (GTX629630, GeneTex, Irvine, CA, USA). Blots were washed three times with TPBS containing 0.05% Tween-20 and incubated with horseradish peroxidase (HRP)-conjugated secondary antibody (1:5000) (Santa Cruz, Santa Cruz, CA, USA) for 1 h at room temperature. Signals were visualized with enhanced chemiluminescence (Thermo Scientific) and imaged using a Bio-Rad ChemiDoc XRS^+^ system (Bio-Rad Laboratories, Inc., Hercules, CA, USA) after an additional wash.

### 4.8. Histopathological Assessment with Hematoxylin and Eosin Staining

Lung tissues were fixed in 4% paraformaldehyde and then embedded in paraffin, cut into 4-µm sections, and stained with hematoxylin and eosin (HE). The pathological changes in lung tissues were observed using an optical microscope. The histological scoring parameters included edema, alveolar and interstitial inflammation, alveolar and interstitial hemorrhage, atelectasis, and hyaline membrane formation. The score of each item was recorded as one of the following four grades: no injury scored 0, injury in 25% of the field scored 1, injury in 50% of the field scored 2, injury in 75% of the field scored 3, and injury throughout the field scored 4. Maximum possible score was 28 [13,46].

### 4.9. Statistical Analyses

All values were expressed in mean ± standard error (SEM), with differences between groups being analyzed using one-way analysis of variance (ANOVA), followed by post hoc Tukey’s multiple comparisons test, and the results were considered statistically significant at *p* < 0.05.

## 5. Conclusions

**Figure 7:**
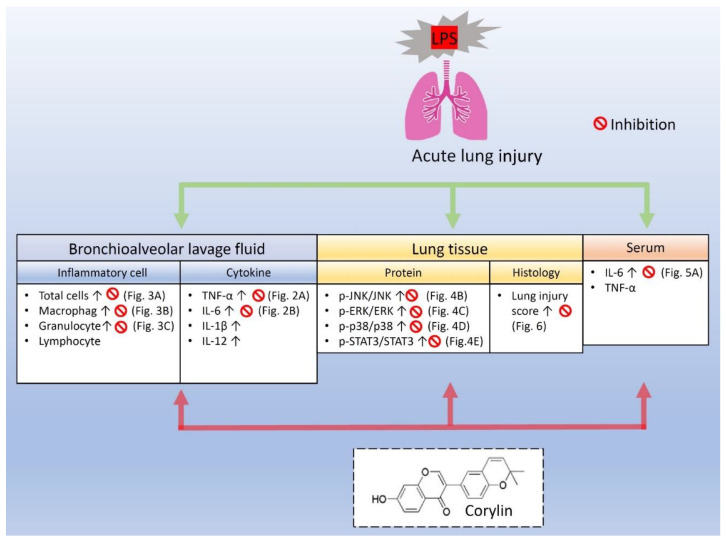
Anti-inflammatory effect of corylin on LPS-induced ALI. The experimental results demonstrated that corylin attenuated the overproduction of IL-6 in LPS-activated human bronchial epithelial cells. In intratracheal LPS-induced ALI mice, corylin attenuated tissue damages, suppressed inflammatory cell infiltration, and decreased secretion of IL-6 and TNF-α in the BALF and serum; moreover, it further inhibited the expression of phosphorylation of mitogen-activated protein kinases (MAPKs), including the expression of p-JNK/JNK, p-ERK/ERK, p-p38/p38, and repressed the activation of signal transducer and activator of transcription 3 (STAT3) in lung. Taken together, our results are the first to demonstrate the anti-inflammatory effects of corylin on LPS-induced ALI and suggest corylin has significant potential as a novel therapeutic agent for ALI.

To our knowledge, this study is the first report to demonstrate the anti-inflammatory effects of corylin on LPS-induced ALI mice, evidenced by the reduction in inflammatory cell infiltration and decrease in inflammatory cytokines through inhibiting MAPKs and IL-6/STAT3 signaling pathways (Figure 7). These results suggest that corylin, a natural compound isolated from *Psoralea corylifolia* L., could be a novel effective drug for ALI treatment.

## Supplementary Materials

The following are available online at https://www.mdpi.com/article/10.3390/ph14101046/s1, Figure S1: Flow cytometric analysis for immune cell subset identification in BALF.
