# Supplementary material for: Corylin Ameliorates LPS-Induced Acute Lung Injury via Suppressing the MAPKs and IL-6/STAT3 Signaling Pathways
Source: Pharmaceuticals (Basel). 2021 Oct 14;14(10):1046. doi: 10.3390/ph14101046 (PMC8537250; doi:10.3390/ph14101046)
Supplement: Supplementary file 1 [file pharmaceuticals-14-01046-s001.zip › pharmaceuticals-1418206-supplementary.pdf]

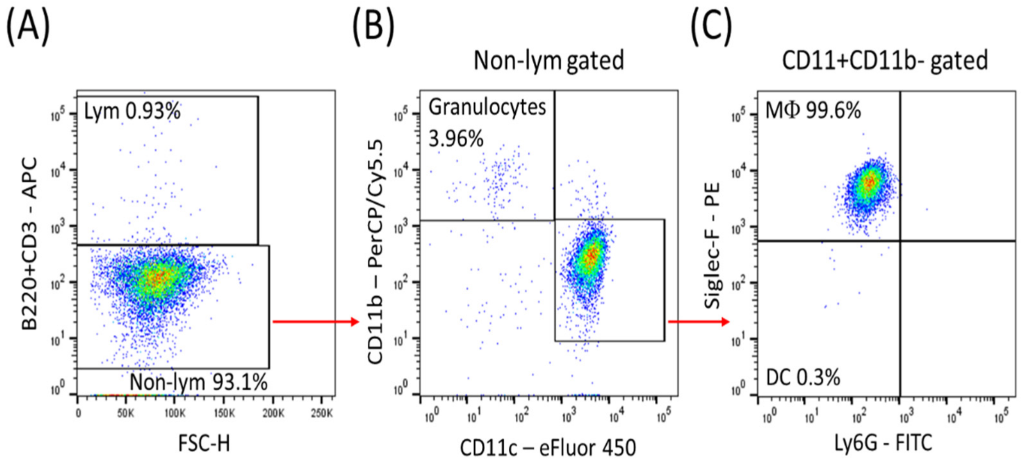

Supplemental Figure S1. Flow cytometric analysis for immune cell subset identification in BALF. Representative dot plots from a LPS-tread mouse are shown for gating for (A) lymphocytes (Lym; $CD3^{+}$ or $B220^{+}$ cells) among viable cells. (B) Granulocytes ($CD11c^{-}CD11b^{+}$) are gated from non-lymphocytes. (C) Macrophages (MΦ; $Ly6G^{-}Siglec\text{-}F^{+}$) or dendritic cells (DC; $Ly6G^{-}Siglec\text{-}F^{-}$) are gated from $CD11c^{+}CD11b^{-}$ cells.
